# Enhanced chlorhexidine skin penetration with eucalyptus oil

**DOI:** 10.1186/1471-2334-10-278

**Published:** 2010-09-22

**Authors:** Tarja J Karpanen, Barbara R Conway, Tony Worthington, Anthony C Hilton, Tom SJ Elliott, Peter A Lambert

**Affiliations:** 1 Life & Health Sciences, Aston University, Aston Triangle, Birmingham, UK; 2 Department of Clinical Microbiology, University Hospitals Birmingham NHS Foundation Trust, Queen Elizabeth Hospital, Edgbaston, UK; 3 Pharmacy and Pharmaceutical Sciences, University of Huddersfield, Huddersfield, UK

## Abstract

**Background:**

Chlorhexidine digluconate (CHG) is a widely used skin antiseptic, however it poorly penetrates the skin, limiting its efficacy against microorganisms residing beneath the surface layers of skin. The aim of the current study was to improve the delivery of chlorhexidine digluconate (CHG) when used as a skin antiseptic.

**Method:**

Chlorhexidine was applied to the surface of donor skin and its penetration and retention under different conditions was evaluated. Skin penetration studies were performed on full-thickness donor human skin using a Franz diffusion cell system. Skin was exposed to 2% (w/v) CHG in various concentrations of eucalyptus oil (EO) and 70% (v/v) isopropyl alcohol (IPA). The concentration of CHG (μg/mg of skin) was determined to a skin depth of 1500 μm by high performance liquid chromatography (HPLC).

**Results:**

The 2% (w/v) CHG penetration into the lower layers of skin was significantly enhanced in the presence of EO. Ten percent (v/v) EO in combination with 2% (w/v) CHG in 70% (v/v) IPA significantly increased the amount of CHG which penetrated into the skin within 2 min.

**Conclusion:**

The delivery of CHG into the epidermis and dermis can be enhanced by combination with EO, which in turn may improve biocide contact with additional microorganisms present in the skin, thereby enhancing antisepsis.

## Background

Chlorhexidine (CHG) is a broad spectrum antimicrobial agent widely used for skin antisepsis prior to invasive procedures. However, the efficacy of CHG is reduced in the presence of organic matter and at low pH [1]. Furthermore, CHG, as with other antiseptic preparations exhibits restricted penetration through the skin; our previous studies demonstrate that CHG from aqueous and alcoholic [70% (v/v) isopropyl alcohol (IPA)] solutions poorly penetrate the full thickness skin to the deeper skin layers [2,3]. This limits its efficacy against microorganisms residing in the lower layers of the epidermis and dermis, including hair follicles and sebaceous glands [2-6]. These persisting microorganisms, which include coagulase negative staphylococci, anaerobic bacteria such as *Propionibacterium *spp., and yeast *Candida *spp., may subsequently cause infection when the protective skin barrier is breached during surgical procedures [7-10]. These microorganisms may also contaminate invasive medical devices such as intravascular catheters when they are passed through the skin, and thereby result in infection [11]. This residual source of microorganisms also offers an explanation for the relatively high incidence of surgical site infections which occurs despite the scrupulous use of currently available skin antiseptics. Indeed, an estimated 5% of patients who have undergone a surgical procedure develops a surgical site infection [12]. Novel strategies to enhance the penetration of antiseptic agents into the skin, thereby improving their efficacy against microorganisms located in the epidermis and dermis are therefore needed if these infections are to be prevented

Developments in the transdermal delivery of drugs offer a potential solution to improvement in the penetration of antiseptic agents into the skin. One such approach has been the application of essential oils, such as eucalyptus oil (EO), which contains terpenes [13]. Eucalyptus oil is an effective skin penetration enhancer and it contains 1,8-cineole, a monoterpene cyclic ether, which can enhance penetration of both lipophilic and hydrophilic compounds [14-17]. Terpenes, including 1,8-cineole, bind to the stratum corneum (SC) and are thought to enhance lipophilic drug penetration by increasing the partition coefficient and hydrophilic drug penetration by increasing the diffusion coefficient [18,19]. 1,8-cineole has been found to increase skin penetration by disrupting intercellular lipids in SC and to change SC membrane fluidity at the concentrations as low as 1% to 5% [16,17,19-22]. Yamane *et al*. [17] however showed that the effect of lipid disruption was reversible and that 1,8-cineole did not result in lipid depletion from the SC.

Essential oils have also a broad spectrum of antimicrobial activity and this property has been harnessed in therapeutics, including skin cleansing (MRSA decolonisation) and treatment of necrotic ulcers [23-25]. Eucalyptus oil may therefore serve as a suitable candidate for enhancing the delivery of CHG into the skin, including hair follicles and sebaceous glands, where many microorganisms reside. The presence of EO may also enhance the antimicrobial activity of CHG, as the combination has been shown to have synergistic antimicrobial activity against bacteria [26]. The aim of the current study was to evaluate the skin penetration of CHG and its retention at various depths of skin in the presence of EO.

## Methods

### Materials

Sodium heptane sulphonate, diethylamine (both high-performance liquid chromatography [HPLC] grade), phosphate buffered saline (PBS) tablets, aqueous 20% (w/v) CHG, eucalyptus oil (EO) (containing 82.9% cineole) and isopropyl alcohol (IPA) were purchased from Sigma-Aldrich (Dorset, UK). Phosphate buffered saline (Sigma-Aldrich, Dorset, UK) was reconstituted from tablets according to manufacturers' instructions. Methanol and glacial acetic acid (all HPLC grade) were purchased from Fisher Scientific (Leicestershire, UK).

### Skin samples

Full thickness human skin samples were obtained from three patients undergoing breast reduction surgery and who consented their excised skin for ethically approved research study. The donor skin was frozen on the day of excision and stored at -70°C until required. Full ethical committee approval was obtained prior to this study from South Birmingham Research Ethics Committee.

### Quantification of CHG

High-performance liquid chromatography was used to measure CHG in the skin samples obtained during the penetration studies. The analyses were performed with an Agilent 1200 series HPLC system (Agilent Technologies, UK). The samples were run at a flow rate of 1.2 mL/min at room temperature through a reverse phase chromatography column [CPS-2 Hypersil; dimension 150 × 4.6 mm, 5 μm particle size (Thermo Electron Corporation, UK)] with ultraviolet detection at a wavelength of 254 nm. The isocratic mobile phase consisted of a methanol: water mixture (75:25) with 0.005 M sodium heptane sulphonate and 0.1% (v/v) diethylamine adjusted to pH 4.0 with glacial acetic acid. The method for CHG quantification by HPLC was validated and the levels of detection (LOD) and quantification (LOQ) were determined as previously described [2].

### CHG skin penetration profile studies

Skin penetration studies were performed with vertical Franz diffusion cells as previously described [2]. In brief, skin samples were mounted onto Franz diffusion cells with the stratum corneum (SC) uppermost facing the donor compartment. The receptor compartment was filled with 29 mL of PBS maintained at 37°C). The skin was left to equilibrate for 30 min to reach the temperature of 32°C before application of the test solution (the surface area of skin exposed to the test compound was 3.14 cm2).

Twenty percent (w/v) aqueous CHG was diluted with distilled water, IPA and EO to obtain the final concentrations of 2% (w/v) CHG with 5%, 10%, 20% and 50% (v/v) EO and 10% (v/v) EO with 2% (w/v) CHG in 70% (v/v) IPA. Tween 80 [0.1% (v/v)] was added to the test solutions to enhance EO solubility in the vehicle. One mL of each test solution was spread over a separate skin surface in the donor compartment. Following a 2 min, 30 min and 24 h exposure, skin samples were removed, washed with PBS and sprayed with a cryospray (Bright Instruments, Cambridgeshire, UK) and frozen at -20°C. Punch biopsies (7 mm in diameter) were then cut from each frozen sample in triplicate and the samples were sectioned horizontally with a cryotome (Bright Instruments) into 20 μm sections (from the surface to a depth of 600 μm) and 30 μm sections (from depths of 600 to 1500 μm).

Chlorhexidine was extracted from the skin by mobile phase solution and analysed by HPLC (CHG extraction was validated prior to the study as described previously) [2]. The amounts of CHG were calculated as μg per mg of skin. Control skin (with no added CHG treatment) was analysed parallel to the test samples. The assay was performed in triplicate.

### Determination of CHG permeation through the full thickness skin

During the skin permeation studies 1 mL of receptor fluid was removed every 30 min for 2 h, every hour between 2 to 6 h and at 8 h, 12 h and 24 h. Fluid removed from the receptor compartment was immediately replaced with an equal volume of fresh PBS solution. All samples were filtered and analysed by HPLC. The assays were performed in triplicate.

### Statistical analysis

The data obtained were analysed by a student t-test with INSTAT3 software (Graph pad software version 3.06) with a p < 0.05 level of significance.

## Results

### Chlorhexidine skin penetration following exposure to CHG in combination with 50% (v/v) EO

**Figure 1:**
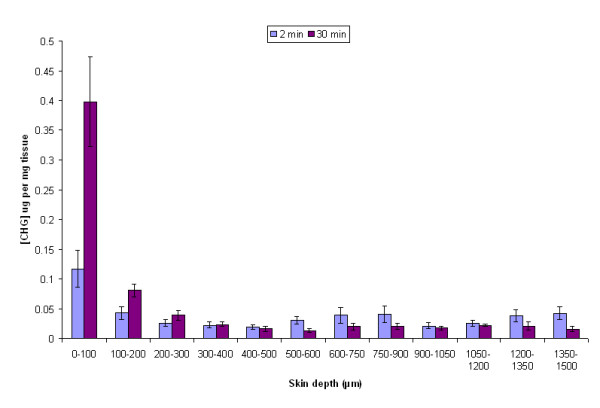
**The amount of CHG (μg/mg of tissue) at increasing depths of excised human skin after a 2-min and 30-min exposure to 2% (w/v) CHG in 50% (v/v) EO (mean ± SEM, n = 15)**.

Combining 2% (w/v) CHG with 50% (v/v) EO enhanced penetration of CHG into the lower layers of skin within 2 min; the CHG concentrations achieved at depths of 300- 1500 μm were between 0.019 - 0.043 μg per mg tissue (Figure 1). After 30 min the concentration of CHG in the upper 100 μm, following application of 2% (w/v) CHG with 50% (v/v) EO, was 0.398 (+/-0.076) μg per mg tissue (Figure 1).

### CHG skin penetration following exposure to CHG in various concentrations of EO

**Figure 2:**
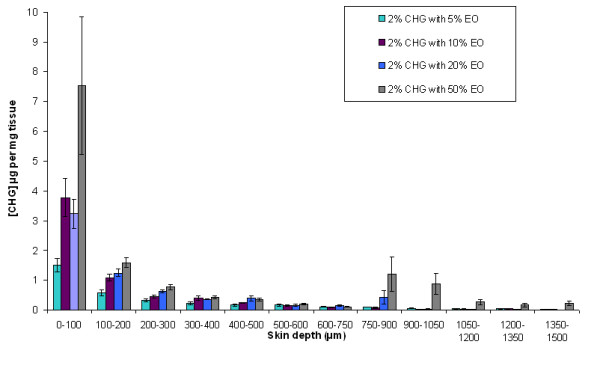
**Penetration profile showing the concentration of CHG (μg/mg of tissue) in excised human skin after 24 hr exposure to 2% (w/v) CHG in combination with various concentrations of EO (mean ± SEM, n = 15)**.

The effect of different EO concentrations on CHG penetration into the skin was evaluated over a 24-h period. Five percent (v/v) EO facilitated greater CHG skin penetration to the deeper layers of the skin (below 300 μm) and 10% (v/v) EO enhanced CHG skin penetration in the upper 900 μm. There were no significant differences in CHG concentration achieved in the skin following application of 2% (w/v) CHG with 10% (v/v) EO or 20% (v/v) EO (Figure 2).

### Chlorhexidine skin penetration with alcohol and EO

**Figure 3:**
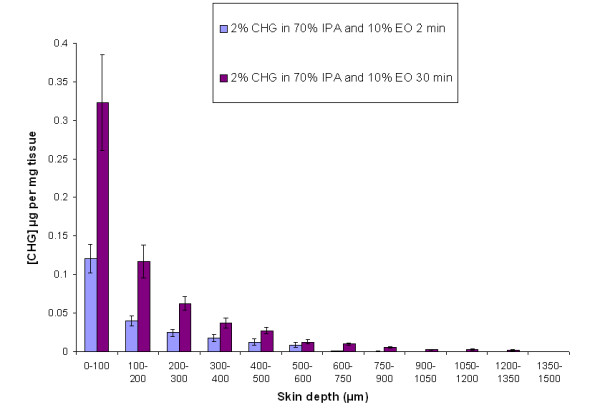
**The amount of CHG (μg/mg of tissue) at increasing depths of excised human skin after a 2-min (n = 10) and 30-min exposure (n = 15) to 2% (w/v) CHG in 70% (v/v) IPA and 10% (v/v) EO (mean ± SEM)**.

The optimum EO concentration, which enhanced CHG penetration into the full thickness human skin, was further evaluated in combination with 2% (w/v) CHG in 70% (v/v) IPA. Ten percent (v/v) EO in combination with 2% (w/v) CHG in 70% (v/v) IPA demonstrated enhanced CHG skin penetration after a 2-min and 30-min exposure on the skin (Figure 3).

### CHG penetration through the full thickness skin

Chlorhexidine was not detected in the receptor compartment (LOD 0.0157 μg/mL) during the 24 h skin permeation study under any conditions when using full thickness human skin (epidermis and dermis) from two of three different donors. Less than 0.0016% of the applied dose of CHG was detected during the skin permeation studies on the third donor skin (< 0.32 μg/mL) following 24-h exposure to 2% (w/v) CHG with 50% (v/v) EO.

## Discussion

In the current study penetration of CHG into the lower layers of the epidermis and dermis was enhanced when applied in combination with EO. This enhanced penetration has the potential for improving skin antisepsis in clinical practice by increasing the concentration of CHG beneath the surface layers of skin where microorganisms can reside. Indeed, the concentration of CHG within the upper 100 μm of skin was increased from 0.023 (+/- 0.007) μg/mg of tissue (as presented in our previous study [3]) to 0.121 (+/- 0.019) μg/mg of tissue after 2 min application of 2% (w/v) CHG in 70% (v/v) IPA and 10% (v/v) EO compared to CHG/IPA alone. Assuming that 1 g of tissue is equal to 1 mL, these concentrations of CHG achieved are higher than the minimum bactericidal concentration against many skin microorganisms such as *Staphylococcus epidermidis *[26]. However, a microbial reservoir can reside in the deeper layers of the skin (and sebaceous glands) following skin antisepsis [4], which may contribute to high number of infections associated with invasive procedures such as surgery or insertion of intravascular catheters. Improved skin delivery of antiseptic agents may therefore enhance skin antisepsis and reduce the risk of infection associated with the invasive procedures and also intravascular catheters. The use of 2% (w/v) CHG in EO may therefore reduce the likelihood of re-seeding of microorganisms onto the skin surface following skin antisepsis, thereby further reducing the risk of infection.

Two percent (w/v) CHG in 70% (v/v) IPA is a recommended antiseptic agent for central venous catheter (CVC) insertion and CVC site care [27]. The skin penetration of CHG following application of 2% (w/v) CHG in 70% (v/v) IPA onto skin was however limited [3]. Chlorhexidine in alcohol has been shown to have superior antimicrobial activity compared to 2% (w/v) aqueous CHG, however their efficacy in reducing intravascular catheter related infections and catheter colonisation are comparable [28-30]. Alcohol, at high concentrations, has a rapid and broad spectrum of antimicrobial activity, but it has been shown to extract SC lipids and dehydrate SC proteins, therefore increasing the SC permeation barrier [31-34]. However, combining 10% (v/v) EO with 2% (w/v) CHG in 70% (v/v) IPA significantly enhanced CHG skin penetration compared to CHG/IPA alone. Similar previous studies on terpenes in combination with ethanol have shown increased skin permeation of diclofenac sodium [35]. The application of CHG in alcohol and EO seems to offer a solution for enhancing CHG skin penetration but still retaining its skin surface antiseptic activity.

Eucalyptus oil and chlorhexidine are currently used in various pharmaceutical preparations, which are applied to the skin and mucous membranes. These compounds are regarded as safe with few reported adverse reactions. Side effects of CHG have included contact dermatitis and rarely hypersensitivity and anaphylaxis. In comparison, EO adverse reactions are less well known, however 7% EO has been used on necrotic neck ulcers (in combination with other oils) without adverse reactions [25]. Regarding systemic reactions, in this current study CHG was only detected at negligible levels in the receptor compartment suggesting that CHG does not permeate through the full skin thickness, and is retained within the tissue. This property reduces the risk of systemic adverse reaction. Indeed, these results further support previous research on another CHG based compound, chlorhexidine phosphanilate, which did not permeate through the full thickness of skin [36]. Furthermore, other studies have shown that cineole, which is the predominant terpene in EO, binds to SC and is retained in the skin and does not permeate through the skin in *in vitro *assays [18,19,37]. It would therefore appear that both CHG and EO have a minimal risk of stimulating systemic reactions as they poorly permeate the whole skin. Further clinical studies on this are however required to substantiate this.

## Conclusions

In conclusion, the current study demonstrates that 2% (w/v) CHG penetration into the deeper layers of skin was significantly enhanced with EO compared to CHG in aqueous solutions or in 70% (v/v) IPA. Furthermore, 2% (w/v) CHG in combination with 70% (v/v) IPA and 10% (v/v) EO significantly increased the amount of CHG in the skin within 2 min compared to CHG/IPA. These exciting results lay the foundation for further research within this area with a view to potentially adopting alternative strategies for enhanced skin antisepsis in clinical practice. Further studies need to be undertaken to determine the skin tolerance of EO and the clinical efficacy of CHG in combination with EO in reducing the number of infections associated with invasive procedures such as insertion of intravascular catheters.

## Competing interests

The synergistic antimicrobial activity of CHG and EO and the enhanced skin permeation of CHG in combination with EO holds an international patent (PCT/GB2008/002832). Other authors have no competing interests.

## Authors' contributions

TJK, TW, BRC and PAL have participated in the design, analysis of the data and drafting this manuscript. TJK performed the skin permeation studies. ACH contributed to the statistical analysis of the data and drafting this manuscript. TSJE contributed to the analysis of the data and drafting this manuscript. All authors have read and approved the final manuscript.

## Pre-publication history

The pre-publication history for this paper can be accessed here:

http://www.biomedcentral.com/1471-2334/10/278/prepub
